# Gene Expression and Secondary Metabolic Regulation Underlying Variegated Leaf Formation in Sweetpotato

**DOI:** 10.3390/plants15152393

**Published:** 2026-08-05

**Authors:** Kangbowen Wang, Peng Li, Genmin Lv, Haojia Zhang, Zhelin Liang, Kai Zhang

**Affiliations:** 1 College of Agronomy and Biotechnology, Southwest University, Beibei, Chongqing 400715, China; 2 Integrative Science Center of Germplasm Creation in Western China (Chongqing) Science City, Southwest University, Beibei, Chongqing 400715, China; 3 Chongqing Key Laboratory of Biology and Genetic Breeding for Tuber and Root Crops, Beibei, Chongqing 400715, China

**Keywords:** sweetpotato, variegated leaves, miRNA, mRNA, anthocyanin, flavonoids, metabolome

## Abstract

Variegated sweetpotato leaves are of interest for ornamental and functional-food applications, yet the molecular and metabolic basis of their coloration remains poorly understood. A sweetpotato accession with variegated leaves was found incidentally in the field, with rare and vivid Pink-purple coloration in the apical leaves. To investigate the mechanism underlying this interesting phenotype, this material was maintained in the laboratory, and variegated and normal green leaves from this plant were analysed by transcriptome profiling, miRNA sequencing and metabolome analysis. By integrating differential mRNA expression analysis, target-gene prediction, miRNA sequencing and metabolite identification, we screened candidate miRNA-mRNA modules associated with this leaf-colour formation. Using FDR < 0.05 and |log_2_FC| ≥ 1 as thresholds, 49 differentially expressed miRNAs and 692 regulatory relationships involving differentially expressed target mRNAs associated with these miRNAs were identified. Functional annotation indicated that the iba_638_x1- G10133|TU16655 and iba_730_x1- G3592|TU5891 modules were associated with anthocyanin/flavonoid biosynthesis and vacuolar transport, respectively. Metabolome analysis showed that the total relative abundance of flavonoids in Pink leaves was 2.98-fold higher than that in CK, and 28 flavonoids accumulated to more than 2-fold higher levels in Pink than in CK. The differentially accumulated metabolites mainly included anthocyanin glycosides, such as petunidin-3-O-glucoside, delphinidin-3-O-glucoside and cyanidin-3,5-O-diglucoside, and flavonol glycosides, such as quercetin-3-O-sophoroside, isorhamnetin-3-O-glucoside and kaempferol-3-O-sophoroside-7-O-glucoside. These results indicate that the variegated phenotype of Pink leaves is mainly associated with differential accumulation of anthocyanin glycosides and flavonol glycosides.

## 1. Introduction

Sweetpotato (*Ipomoea batatas* (L.) Lam.) is a vegetatively propagated storage-root crop cultivated for food, feed, industrial uses, edible leaves, and ornamental foliage. Its broad environmental adaptability and extensive root and shoot diversity make it important for crop production and germplasm improvement. Unlike ordinary green-leaf cultivars, ornamental or variegated sweetpotato has distinctive value for landscape planting and functional food development because its leaves display diverse colours, including purple-red, golden yellow and variegated patterns. Leaf colour reflects the relative abundance and spatiotemporal distribution of pigments such as chlorophylls, carotenoids and anthocyanins [1]. In purple-leaf sweetpotato, anthocyanins accumulate extensively in leaves and are accompanied by broad activation of the flavonoid metabolic pathway [2]. Anthocyanins are major plant secondary metabolites. Their biosynthesis starts from phenylalanine and proceeds through chalcone synthase (CHS), chalcone isomerase (CHI) and flavanone 3-hydroxylase (F3H) to generate dihydroflavonol intermediates. These intermediates enter the anthocyanin-specific pathway through dihydroflavonol 4-reductase (DFR) and are ultimately converted by enzymes such as anthocyanidin synthase (ANS) into diverse anthocyanin glycosides [2,3,4,5,6,7]. At the transcriptional level, the expression of anthocyanin biosynthetic genes is tightly regulated by transcription-factor networks, with the MYB-bHLH-WD40 (MBW) complex as a central module. In Arabidopsis, anthocyanin biosynthesis is mainly regulated by an MBW complex composed of MYB transcription factors (PAP1/PAP2), bHLH proteins (TT8/EGL3/GL3) and the WD40 protein TTG1, which jointly activate downstream structural genes such as *DFR* and *ANS* [3,4]. In monocots such as maize, the MYB factors C1/Pl1 cooperate with the bHLH factors R/B and the WD40 protein PAC1 to regulate tissue-specific deposition of anthocyanin pigments [8,9]. In rice, hierarchical regulation among MBW components has been reported, with the bHLH factor S1 sequentially activating C1 (MYB) and WA1 (WD40) [10]. In tomato, SlAN2 (MYB), SlJAF13/SlAN1 (bHLH) and SlAN11 (WD40) jointly fine-tune flavonoid and anthocyanin accumulation [11,12,13,14]. In sweetpotato, IbMYB1 has been identified as an activator in purple-fleshed cultivars [6], but the molecular mechanisms by which the MBW complex regulates leaf-colour formation and secondary metabolism remain largely unclear.

MicroRNAs (miRNAs) are endogenous non-coding RNAs of approximately 21–24 nucleotides and are important post-transcriptional regulators in plants. They usually pair with target mRNAs to trigger target-transcript cleavage or translational repression, thereby participating in plant growth, development, stress responses and secondary-metabolism regulation [15,16]. Increasing evidence indicates that miRNAs regulate anthocyanin and flavonoid metabolism. miR156 negatively regulates anthocyanin accumulation by targeting SPL transcription factors [17]. miR858a targets R2R3-MYB transcription factors; overexpression of miR858a in Arabidopsis downregulates multiple MYB transcription factors that regulate flavonoid biosynthesis [18]. Target prediction for miRNAs in the medicinal plant Podophyllum hexandrum suggested that miR5532 may target 2-hydroxyisoflavanone dehydratase in isoflavone biosynthesis, miR829.1 may target chalcone synthase to regulate flavonoid biosynthesis, miR1873 may target dihydroflavonol 4-reductase C in flavonoid biosynthesis, and miR1438 may regulate caffeoyl-CoA O-methyltransferase, thereby affecting secondary metabolic pathways including phenylalanine metabolism, phenylpropanoid biosynthesis and flavonoid biosynthesis [19]. Evidence from other species further supports a role for miRNA modules in pigment regulation: miR828/miR858 regulate anthocyanin and flavonol accumulation in grape, and miR156-SPL networks influence anthocyanin biosynthesis in poplar [20,21]. However, these studies concern pigment accumulation rather than stable sectorial variegation; therefore, the contribution of miRNAs to the sweetpotato phenotype examined here remains a testable hypothesis.

**Figure 1:**
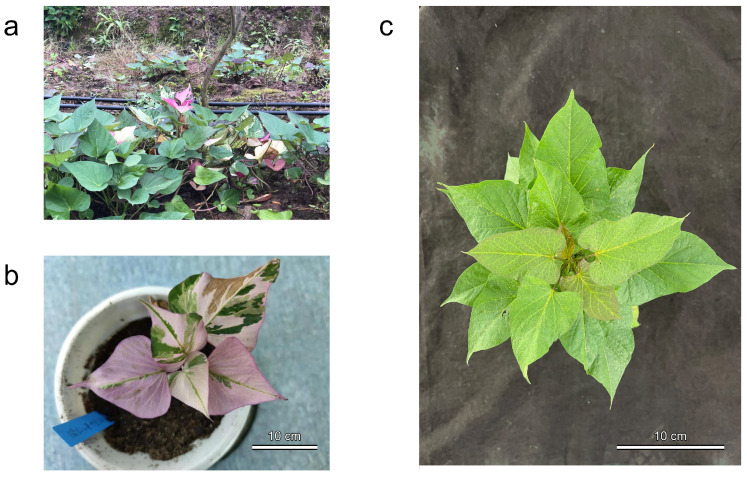
Leaf-colour phenotypes in the field and the growth of variegated sweetpotato in chamber. (**a**), The variegated sweetpotato plant observed in the field conditions. (**b**), Apical part of the plant growing in the chamber exhibited variegated-leaf phenotype. (**c**), The bottom parts of the plant growing in the chamber exhibited normal green-leaf phenotype.

We incidentally identified a variegated sweetpotato plant in the field whose apical leaves showed rare, vivid Pink-purple coloration (Figure 1a). To investigate the possible mechanism underlying this phenotype, the plant was transferred to the laboratory and its variegated leaves and normal green leaves were used for high-throughput transcriptome sequencing, small RNA sequencing and metabolome analysis. Differentially expressed miRNAs and their target mRNAs were identified, and an miRNA-mRNA regulatory network was constructed to screen key genes associated with anthocyanins, flavonoids and variegated leaf-colour formation. The resulting multi-omics framework was intended to generate testable candidates for subsequent genetic and functional validation.

## 2. Materials and Methods

### 2.1. Plant Materials and Sampling

The plant material used in this study was derived from naturally occurring variegated plants of the sweetpotato cultivar Yuhongxinshu 4 (渝红心薯4号). On 28 October 2020, approximately three plants exhibiting variegated leaves were identified in a field-grown population of Yushu 33 at Beibei, Chongqing. These plants were selected as the source material for subsequent cultivation and sampling. The present study focused on the naturally occurring variegated phenotype identified in Yushu 33. The plants were then transferred to a growth chamber maintained at 25 °C under a 14 h light/10 h dark photoperiod, 50% relative humidity, and a light intensity of 150 μmol m^−2^ s^−1^ at the Science and Technology Building of Southwest University, Chongqing, China. For RNA sequencing, normal green leaves (CK) and variegated leaves (Pink) were collected from three biological replicates per group (Figure S1).

### 2.2. RNA Sequencing

After total RNA extraction, all samples were sent to Novogene Bioinformatics Technology Co., Ltd. (Tianjin, China) for sample quality assessment, library construction and sequencing. RNA quality control included preliminary assessment of RNA integrity by agarose gel electrophoresis, RNA purity measurement using NanoDrop 2000 spectrophotometer (Thermo Fisher Scientific, Waltham, MA, USA), accurate quantification of RNA concentration using Qubit 2.0 and further assessment of RNA integrity using an Agilent 2100 Bioanalyzer (Agilent Technologies, Santa Clara, CA, USA). Samples that passed quality control were used for library construction.

After library construction, libraries were preliminarily quantified using Qubit 2.0 Fluorometer. After dilution, insert size was assessed using an Agilent 100 system. When insert size met the expected range, the effective library concentration was accurately quantified by qPCR. Qualified libraries were pooled according to effective concentration and the target data volume, clustered on a cBot Cluster Generation System and sequenced on an NovaSeq 6000 Sequencing System (Illumina, Inc., San Diego, CA, USA).

Raw reads were subjected to quality control after sequencing. During this process, reads containing adapters were removed. Reads were also discarded if unknown bases (N) accounted for more than 10% of the read or if low-quality bases (Q ≤ 5) accounted for more than 50% of the read. The resulting high-quality clean reads were used for subsequent small RNA identification and expression analysis (Table S1).

### 2.3. Differential Expression Analysis of miRNAs

Differential expression analysis was performed in R version 4.6.1 using edgeR version 4.10.1 [22]. The CK and Pink count matrices were converted into DGEList objects for mRNA and miRNA analyses. The group factor was specified with CK as the reference level and Pink as the comparison level, and the design matrix was constructed using model.matrix(~group). Lowly expressed features were filtered using filterByExpr according to the two-group design, resulting in the retention of 36,881 mRNA features and 151 miRNAs. Library sizes were recalculated after filtering, and compositional differences among samples were normalised using the trimmed mean of M-values (TMM) method [23]. Common and tagwise dispersions of the negative binomial model were estimated, after which the edgeR exact test for two-group comparisons was used to assess differential expression between Pink and CK. Raw *p* values were adjusted for multiple testing using the Benjamini–Hochberg procedure [24]. Features with an FDR < 0.05 and |log_2_FC| ≥ 1 were considered significantly differentially expressed. The log_2_FC was defined as log_2_ (Pink/CK), such that positive values indicated upregulation in Pink and negative values indicated downregulation in Pink. Significantly differentially expressed miRNAs were subsequently used for target-mRNA matching and integrated miRNA-mRNA analysis.

### 2.4. Differential Expression Analysis of mRNAs

Differential mRNA expression analysis was performed using gene count matrices from CK and Pink, with statistical testing conducted in edgeR [22]. Differentially expressed mRNAs were screened using FDR < 0.05 and |log_2_FC| ≥ 1 as thresholds. They were then classified as upregulated or downregulated according to the direction of expression change and included in the subsequent integrated miRNA-mRNA analysis.

To improve the interpretability of the integrated analysis, edgeR results for differentially expressed mRNAs were combined with functional annotation tables to extract the annotation name, chromosomal position, log_2_FC and FDR of candidate target transcripts. Inverse expression relationships associated with anthocyanin/flavonoid biosynthesis and flavonoid transport were then prioritised.

### 2.5. Metabolite Identification and Relative-Abundance Description

For metabolomic analysis, samples 1-1, 1-2, and 1-3 were collected from a purple leaf, a pink leaf, and a central variegated leaf, respectively (Figure S2). Metabolite data were obtained from matches against a laboratory database and public databases. According to the annotation using, the metabolites were defined as the following types: 1, phenolic acids; 2, flavonoids; 3, glucosinolates; 4, lipids; and 5, other hydroxycinnamic-acid-related compounds.

### 2.6. Materials and Methods: Gene Ontology Over-Representation Analysis

Gene Ontology (GO) over-representation analysis [25] was performed separately for all differentially expressed transcripts and for the upregulated and downregulated subsets. Differential expression was defined from the edgeR results for Pink relative to CK using an FDR < 0.05 and |log_2_FC| ≥ 1. Transcript-unit (TU) identifiers were extracted from the differential-expression table and matched to the GO associations in iba.annot. When a TU identifier occurred in more than one row, the record with the lowest edgeR FDR was retained; ties were resolved by retaining the record with the larger absolute log_2_FC and then the earlier source row. GO identifiers, term names and ontology assignments were obtained from the GO Basic ontology release dated 8 December 2020.

The statistical background comprised 9021 unique TU identifiers that were present in the differential-expression table and had at least one valid GO annotation. This yielded 2430 mapped differentially expressed transcripts, including 1183 upregulated and 1247 downregulated transcripts. GO terms annotated to 10–500 background transcripts were eligible for testing. For each term, over-representation was assessed using a one-sided hypergeometric test in R 4.6.1. *p* values were adjusted using the Benjamini–Hochberg procedure [24] separately for the complete, upregulated and downregulated transcript sets. The respective corrections included all size-eligible terms with at least one overlapping differentially expressed transcript (527, 487 and 494 terms, respectively). GO terms with an adjusted *p* value < 0.05 were considered significantly enriched, and output tables reported terms represented by at least two differentially expressed transcripts.

For visualisation, the eight highest-ranked terms within each GO domain (biological process, cellular component and molecular function) were selected according to the adjusted *p* value. In the enrichment dot plot, point size represents the number of differentially expressed transcripts assigned to a term, the *x*-axis represents the proportion of the term’s annotated background transcripts that were differentially expressed (DEG count/background count), and colour represents the adjusted *p* value.

## 3. Results

### 3.1. Phenotypes of Variegated and Green-Leaf Sweetpotato

In the field, the apical leaves showed bright Pink to purple colours, while the bottom leaves showed normal green colours. After the material was transferred to the laboratory, apical variegated sectors and normal green-leaf sectors were separately planted in pots. After a period of growth, the variegated leaves (Pink) showed interlaced Pink and green sectors, and the Pink-purple coloration was mainly distributed in parts of the mesophyll. The green leaves (CK) continued to grow normally. The occurrence of Pink-purple sectors in Pink leaves may be associated with the accumulation of coloured metabolites such as anthocyanins, whereas the retention of green sectors indicates that a chlorophyll background remained. To clarify the metabolic basis of the colour difference between Pink and CK, metabolome analysis was subsequently performed to compare metabolite composition and abundance between the two materials.

### 3.2. Flavonoid Accumulation in Pink Leaves

Metabolome analysis identified 40 flavonoid compounds. The total relative abundance of flavonoids in Pink leaves was 1.30 × 10^8^, compared with 4.35 × 10^7^ in green CK leaves, indicating that the abundance in Pink was approximately 2.98-fold higher than that in CK. Among the identified flavonoids, 28 accumulated to more than 2-fold higher levels in Pink than in CK, whereas only three were present at less than 0.5-fold the level in CK. These results indicate an overall trend towards flavonoid accumulation in leaves of Pink.

**Figure 2:**
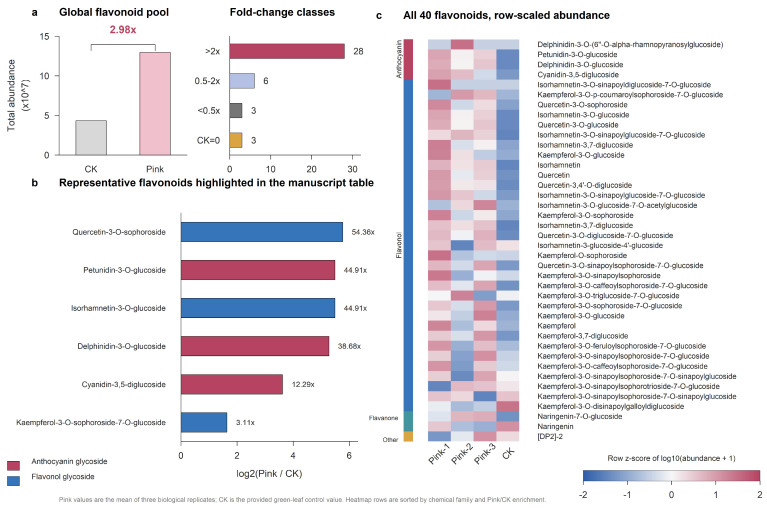
Flavonoid remodelling associated with the Pink-variegated phenotype in sweetpotato leaves. (**a**) Total flavonoid abundance and fold-change distribution of detected flavonoid metabolites in Pink compared with CK samples. The value indicates the fold change in the global flavonoid pool. (**b**) Representative flavonoid metabolites with pronounced enrichment in Pink samples, shown as log_2_-transformed Pink/CK ratios. Colours denote anthocyanin and flavonol glycoside classes. (**c**) Row-scaled heatmap of all detected flavonoids across Pink biological replicates and the CK control, with metabolites grouped by chemical family.

Further comparison showed that several anthocyanins and flavonol glycosides accumulated to higher levels in Pink leaves. The relative abundance of quercetin-3-O-sophoroside in Pink was 3.84 × 10^6^, 54.36-fold higher than in CK. Petunidin-3-O-glucoside and isorhamnetin-3-O-glucoside each accumulated to 44.91-fold higher levels, delphinidin-3-O-glucoside accumulated to a 38.68-fold higher level, and cyanidin-3,5-O-diglucoside accumulated to a 12.29-fold higher level. The relative abundance of kaempferol-3-O-sophoroside-7-O-glucoside in Pink reached 2.04 × 10^7^, approximately 3.11-fold higher than in CK (Figure 2).

High accumulation of anthocyanin glycosides, including petunidin, delphinidin and cyanidin derivatives, may directly influence red-to-purple leaf coloration. Changes in quercetin, kaempferol and isorhamnetin derivatives may also contribute to leaf-colour modulation. Together with the field phenotype, these results indicate that anthocyanin glycoside accumulation might be an important biochemical basis of the Pink-purple sectors of Pink leaves.

### 3.3. Differential Expression of Flavonoid-Biosynthesis Genes

**Figure 3:**
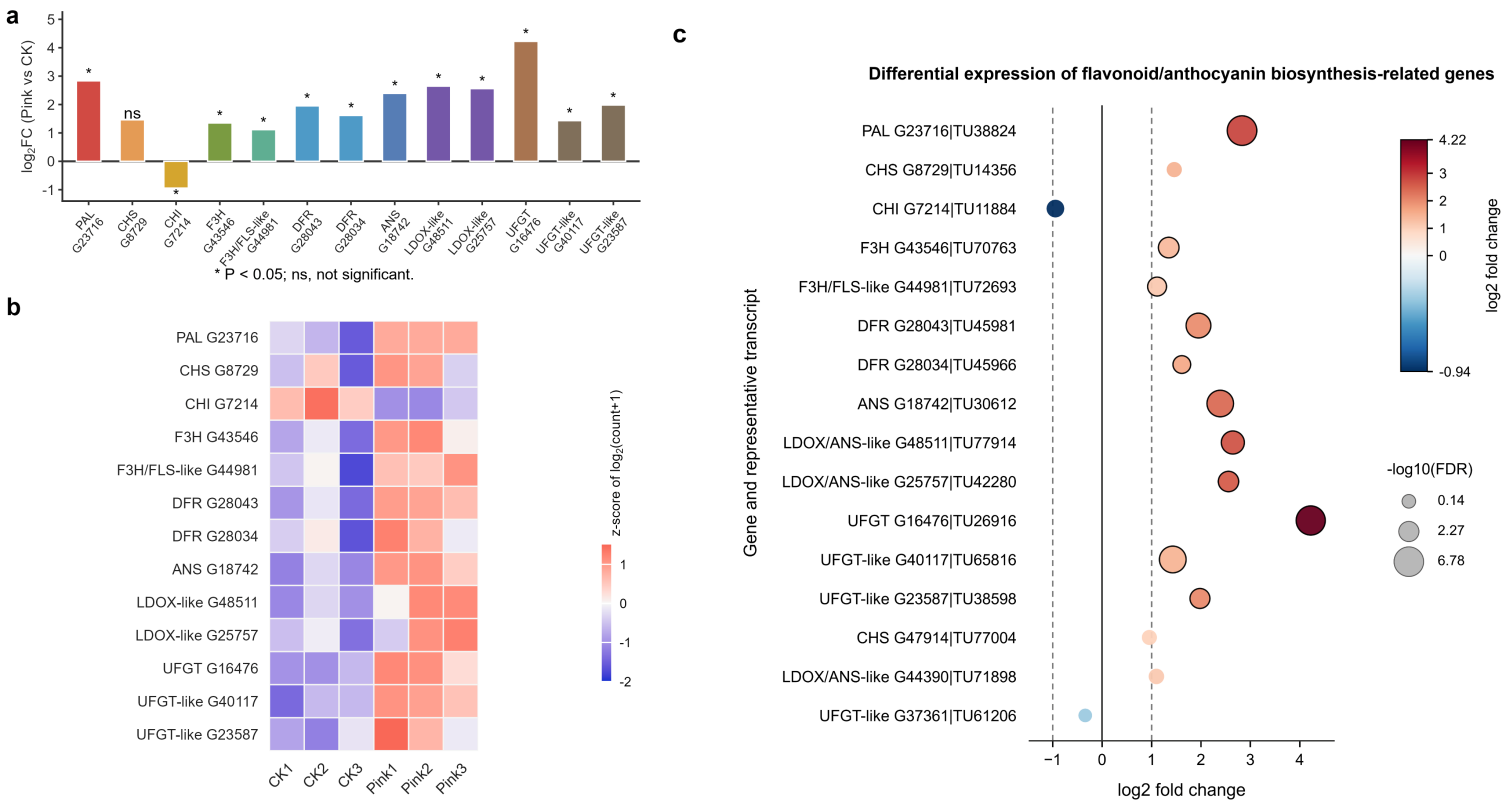
Differential regulation of flavonoid and anthocyanin biosynthetic genes in Pink-variegated leaves. (**a**) Relative expression of representative flavonoid-biosynthesis genes in Pink versus CK samples. (**b**) Z-score-normalised expression heatmap of flavonoid-biosynthesis-related genes across CK and Pink replicates. (**c**) Bubble plot summarising differential expression of flavonoid- and anthocyanin-biosynthesis-related genes. Bubble size denotes −log10 (FDR), and colour indicates expression status.

Transcriptome analysis identified differential expression across several stages of the flavonoid and anthocyanin pathways (Figure 3). Differential expression was defined using FDR < 0.05 and |log_2_FC| > 1.

The phenylalanine ammonia-lyase gene G23716|TU38824 was upregulated in Pink, with a log_2_FC of 2.833 and an FDR of 1.67 × 10^−7^. None of the four detected chalcone synthase candidates met both differential-expression thresholds. G8729|TU14356 had a log_2_FC of 1.461 but an FDR of 0.265.

The chalcone isomerase gene G7214|TU11884 was downregulated, with a log_2_FC of −0.943 and an FDR of 0.043. Its fold change did not reach the predefined magnitude threshold.

The F3H gene G43546|TU70763 and the F3H/FLS-like gene G44981|TU72693 were upregulated. Their log_2_FC values were 1.350 and 1.114, respectively. The corresponding FDR values were 0.0036 and 0.0160.

Two DFR candidates were also upregulated in Pink. G28043|TU45981 and G28034|TU45966 had log_2_FC values of 1.952 and 1.615, respectively. The ANS gene G18742|TU30612 had a log_2_FC of 2.392. Two LDOX-like genes, G48511|TU77914 and G25757|TU42280, had log_2_FC values of 2.647 and 2.559.

Genes associated with anthocyanin glycosylation showed some of the largest expression differences. The UFGT candidate G16476|TU26916 was upregulated, with a log_2_FC of 4.223 and an FDR of 3.06 × 10^−7^. Two additional candidates, G40117|TU65816 and G23587|TU38598, had log_2_FC values of 1.431 and 1.982.

Overall, the largest expression increases were concentrated among middle- and late-stage pathway genes. These included F3H, DFR, ANS/LDOX and UFGT, whereas the detected CHS candidates did not pass both thresholds (Figure 3a–c).

### 3.4. Differential Expression of Transcription-Factor Families

Table 1 summarises the expression patterns of MYB, bHLH and WD40/WD-repeat family members. Both upregulated and downregulated genes were detected in all three families.

Among 79 annotated MYB genes, 23 had FDR < 0.05. Twenty genes also met the |log_2_FC| > 1 threshold, including eight upregulated and twelve downregulated genes. G4796|TU7910 and G31723|TU52014 were upregulated, with log_2_FC values of 2.677 and 2.519.

Among 114 annotated bHLH genes, 32 had FDR < 0.05. Twenty-seven also met the fold-change threshold, including ten upregulated and seventeen downregulated genes. Two bHLH79 candidates had log_2_FC values of 5.602 and 4.244. The bHLH18-like gene G5580|TU9213 had a log_2_FC of 4.393.

Among 104 WD40/WD-repeat genes, 28 had FDR < 0.05. Twenty-two also met the fold-change threshold, with eleven upregulated and eleven downregulated genes. G42625|TU69482 and G28128|TU46106 were upregulated, with log_2_FC values of 5.710 and 3.301.

The putative anthocyanin regulator G13144|TU21541 showed a log_2_FC of 6.378 and an FDR of 1.15 × 10^−18^. By contrast, the JAF13 transcription-factor gene G13155|TU21554 was downregulated, with a log_2_FC of −3.108.

### 3.5. Differentially Expressed miRNAs in Pink Leaves

**Figure 4:**
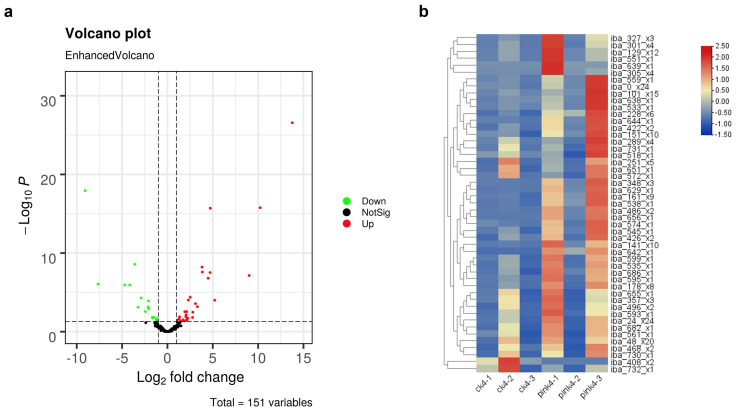
The miRNA expression reprogramming associated with the Pink-variegated phenotype in sweetpotato leaves. (**a**) Volcano plot displaying the differential miRNA expression profile in Pink compared with CK samples. Red and green points represent significantly up- and downregulated miRNAs, respectively; black points indicate miRNAs without significant differential expression. Dashed lines mark the fold-change and significance cut-offs. (**b**) Heatmap showing hierarchical clustering of differentially expressed miRNAs across CK and Pink biological replicates, with colours denoting normalised relative expression levels.

A total of 377 candidate miRNAs were detected across the six expression libraries (Figure 4). Differential-expression analysis was performed for 151 candidate miRNAs. Using FDR < 0.05 and |log_2_FC| > 1, 49 differentially expressed miRNAs were identified. Thirty were upregulated and nineteen were downregulated in Pink (Table S2).

The volcano plot shows differentially expressed miRNAs on both sides of the fold-change distribution (Figure 4a). The number of upregulated miRNAs exceeded the number of downregulated miRNAs. The heatmap shows distinct expression profiles between Pink and CK samples (Figure 4b).

Among the largest changes, iba_327_x3 and iba_639_x1 were upregulated, with log_2_FC values of 13.765 and 10.223. Conversely, iba_357_x3 and iba_574_x1 were downregulated, with log_2_FC values of −9.064 and −7.659.

Target predictions were available for 48 of the 49 differentially expressed miRNAs. These predictions comprised 5533 miRNA–mRNA pairs involving 3252 candidate target genes. Intersection with the differentially expressed mRNA dataset retained 692 predicted associations.

Among these 692 associations, 332 showed opposite expression directions between the miRNA and candidate target transcript. A subset of 195 inverse-expression associations had expectation scores of 4.0 or lower. These associations involved 179 candidate target genes. Iba_327_x3 had fifteen such candidates, whereas iba_101_x15, iba_289_x4 and iba_551_x1 each had ten.

### 3.6. Predicted miRNA–mRNA Associations Related to Flavonoid Metabolism

**Figure 5:**
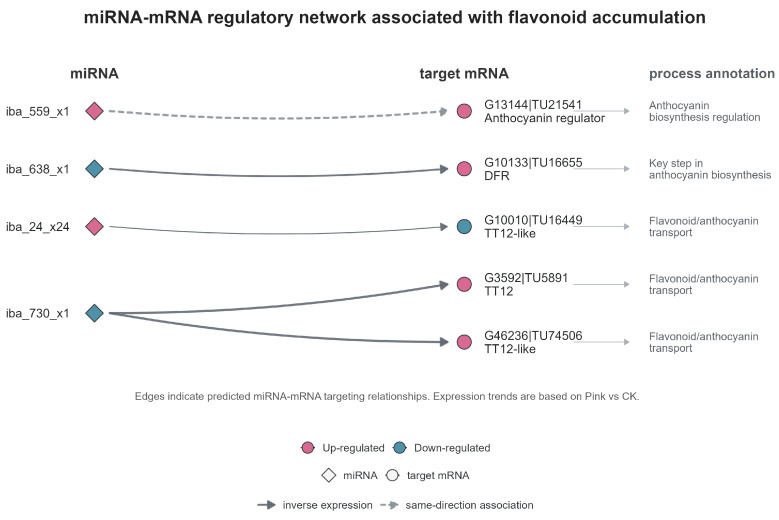
miRNA-mRNA regulatory network associated with flavonoid accumulation in sweetpotato leaves. Predicted regulatory network of differentially expressed miRNAs and their target mRNAs in Pink compared with CK samples. Node colours indicate up- or downregulated expression, and arrows represent predicted miRNA-mRNA targeting relationships related to anthocyanin biosynthesis and flavonoid/anthocyanin transport.

Functional annotation identified 49 differentially expressed mRNAs associated with flavonoid metabolism, anthocyanin biosynthesis, pigment transport or leaf-colour variation. Matching these mRNAs with the 49 differentially expressed miRNAs yielded five predicted associations. These involved four miRNAs and five candidate target transcripts (Figure 5).

Iba_638_x1 was downregulated, whereas its predicted DFR target G10133|TU16655 was upregulated. Iba_730_x1 was downregulated, whereas its predicted TT12/TT12-like targets G3592|TU5891 and G46236|TU74506 were upregulated.

Iba_24_x24 was upregulated, whereas its predicted TT12-like target G10010|TU16449 was downregulated. By contrast, iba_559_x1 and its predicted anthocyanin-regulator target G13144|TU21541 were both upregulated.

Figure 5 summarises these five computationally predicted associations. The directions of the nodes represent the measured expression changes, whereas the edges represent target predictions rather than experimentally validated interactions.

### 3.7. GO Enrichment Is Confined to the Upregulated Transcript Set

**Figure 6:**
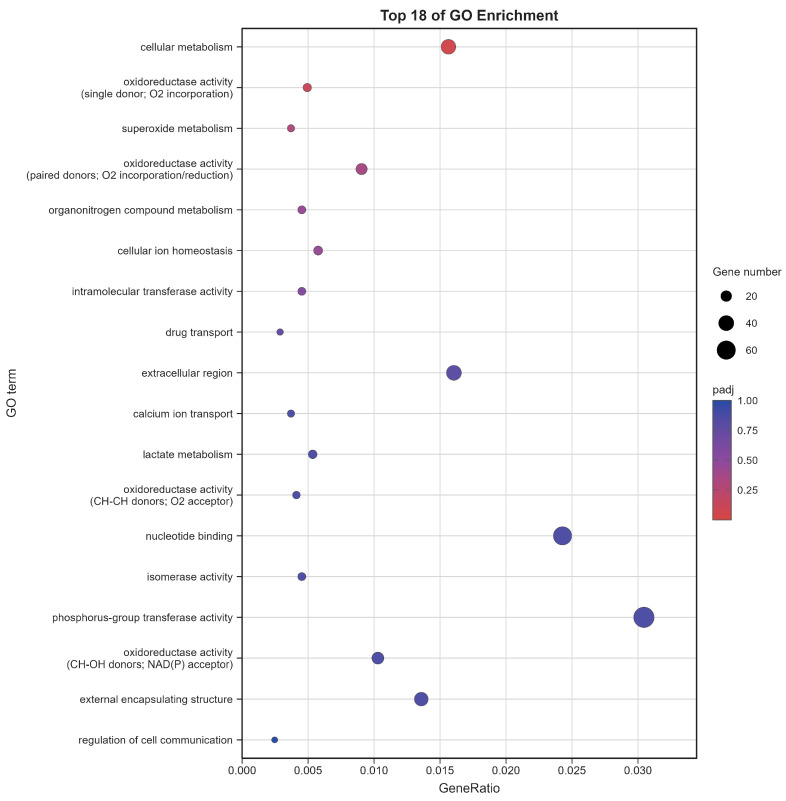
GO enrichment analysis of the top 18 terms. Bubble size represents the number of genes, and colour indicates the adjusted *p*-value.

After correction for multiple testing, significant GO enrichment was detected only in the upregulated transcript set. The molecular-function term “oxidoreductase activity, acting on single donors with incorporation of molecular oxygen” (GO:0016701) contained 11 of the 1183 upregulated transcripts, compared with 18 of the 9021 background transcripts, corresponding to a 4.660-fold enrichment (*p* = 2.485 × 10^−6^; adjusted *p* = 0.00121). The biological-process term “cellular metabolic process” (GO:0044237) contained 24 upregulated transcripts and 82 background transcripts, corresponding to a 2.232-fold enrichment (*p* = 8.759 × 10^−5^; adjusted *p* = 0.01213) (Figure 6).

No GO term remained significant in either the complete differentially expressed transcript set or the downregulated subset. In the complete set, the highest-ranked term was “cellular metabolic process” (38 of 2430 differentially expressed transcripts; 1.720-fold enrichment), but its adjusted *p* value was 0.0638 and therefore did not meet the prespecified significance threshold. These results indicate a direction-specific functional signal associated with upregulated transcripts rather than broad GO enrichment across all differentially expressed transcripts.

## 4. Discussion

Pink leaves showed coordinated changes in anthocyanin and flavonol glycosides. The total flavonoid relative abundance was 2.98-fold higher than that in CK. Similar associations between purple coloration and flavonoid enrichment have been reported in sweetpotato leaves and storage roots [2,26]. Metabolomic comparisons among sweetpotato leaf tips and colour mutants have also linked visible pigmentation to coordinated changes across several flavonoid classes [1,27].

The present phenotype nevertheless differs from uniformly purple tissues described in many previous studies. Pink-purple sectors occurred beside green sectors within the same leaves. The enrichment of petunidin, delphinidin and cyanidin glycosides can account for the red–purple component of this phenotype. Acylation can improve the heat stability of sweetpotato anthocyanins, although the acylation status of the metabolites detected here was not evaluated [26]. The accompanying enrichment of quercetin, isorhamnetin and kaempferol glycosides indicates broader remodelling of the flavonoid profile.

Expression changes were concentrated in the middle and late portions of the anthocyanin pathway. Previous sweetpotato studies have similarly associated purple pigmentation with increased expression of DFR, ANS and UFGT-related genes [5,6,7,27]. In the present study, none of the CHS candidates passed both screening thresholds. By contrast, several F3H, DFR, ANS/LDOX and UFGT candidates were upregulated.

This pattern argues against uniform activation of the entire flavonoid pathway. Instead, it may reflect preferential enhancement of anthocyanin-precursor conversion and subsequent glycosylation. The strong upregulation of G16476|TU26916 provides a potential route for increased anthocyanin glycosylation and stability. However, enzyme activity was not measured, so this interpretation remains based on transcript abundance.

At the broader functional level, the enrichment of GO:0016701 suggests increased representation of oxygen-dependent oxidoreductase functions among transcripts upregulated in Pink leaves. However, the 11 transcripts underlying this term comprised eight lipoxygenase-annotated transcripts and three inositol-oxygenase-annotated transcripts. The signal therefore appears to be driven mainly by lipid-oxidation/oxylipin-related and inositol-oxidation functions and should not be described as direct GO evidence for flavonoid or anthocyanin biosynthesis. It may instead reflect broader oxidative and stress-associated metabolic reprogramming accompanying the Pink-variegated phenotype.

The enrichment of the broad term “cellular metabolic process” is consistent with extensive metabolic adjustment in Pink leaves. Its contributing transcripts include chloroplast- and photosystem-associated proteins, oxidases, glutathione-associated proteins, a shikimate-related protein, and other metabolic proteins. Because this parent term is functionally broad, it supports general metabolic reprogramming but does not identify a specific causal pathway.

Importantly, no phenylpropanoid-, flavonoid- or anthocyanin-specific GO term passed the adjusted-*p*-value threshold. The GO analysis should therefore be presented as contextual support for the transcriptomic and metabolomic evidence, not as independent proof of anthocyanin-pathway activation. The direct upregulation of PAL, F3H, DFR, ANS and UFGT transcripts, together with the accumulation of anthocyanin glycosides, provides more pathway-specific evidence. The GO results add a complementary indication that these changes occur within broader oxidative and cellular metabolic reprogramming. Interpretation should also account for the available annotation coverage: only 9021 of 36,806 unique TU identifiers in the differential-expression table (24.5%) entered the GO background after valid ontology mapping, and the ontology source was the 2020-12-08 GO release.

Anthocyanin transcription is commonly regulated by MYB, bHLH and WD40 proteins. Such regulatory complexes have been characterised in Arabidopsis, tomato and rice [3,4,10,12,14]. The present dataset contained both upregulated and downregulated members of all three families. This bidirectional pattern is more consistent with regulatory-network remodelling than with uniform activation of one transcription-factor family.

G13144|TU21541 was strongly upregulated, whereas a JAF13-like gene was downregulated. In tomato, JAF13 promotes anthocyanin biosynthesis as part of a broader transcriptional network [11]. The opposite expression direction observed here may reflect tissue-specific regulation, functional divergence or incomplete annotation. Family membership and expression direction alone are therefore insufficient to establish the regulatory roles of these sweetpotato genes.

Plant miRNAs frequently reduce target expression through transcript cleavage or translational repression [15,16]. miRNA-dependent regulation of anthocyanin metabolism has also been reported in several species. The miR156–SPL pathway regulates anthocyanin biosynthesis in Arabidopsis and poplar [17,21]. miR828 and miR858 affect anthocyanin and flavonol accumulation in grape, while miR858 regulates the phenylpropanoid pathway in Arabidopsis [18,20].

The inverse-expression patterns of iba_638_x1–DFR and iba_730_x1–TT12/TT12-like genes are compatible with canonical negative miRNA regulation. However, inverse expression does not demonstrate direct targeting. Cleavage assays, reporter assays or genetic perturbation would be required to establish these interactions.

The co-upregulation of iba_559_x1 and G13144|TU21541 does not support a simple direct-repression model. This pattern could result from indirect feedback, temporal separation of expression or differences among cell types. It may also arise from an incorrect target prediction. This pair should therefore remain an unconfirmed candidate association.

The TT12-like candidates provide a possible connection between flavonoid synthesis and intracellular transport. In Arabidopsis, TT12 functions as a vacuolar flavonoid transporter in the seed coat [28,29]. The upregulation of two TT12/TT12-like transcripts coincided with downregulation of iba_730_x1 in Pink. This association is compatible with increased flavonoid transport or sequestration, but transporter activity was not measured. Evidence from Arabidopsis seed tissue cannot establish the same function in sweetpotato leaves.

**Figure 7:**
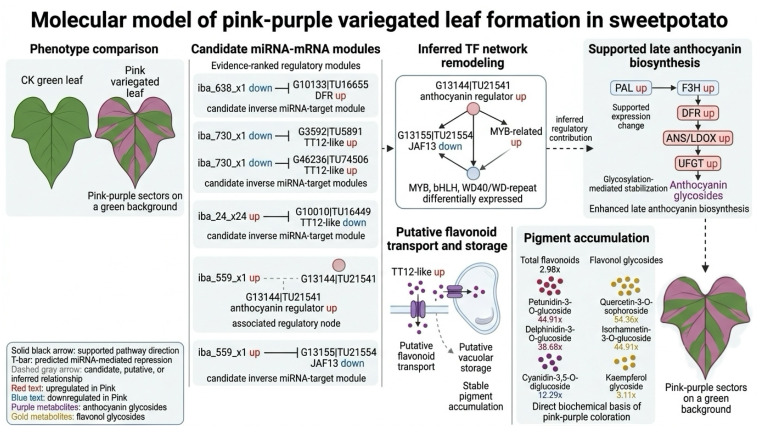
Predicted molecular model of Pink-purple variegated leaf formation in sweetpotato. The model integrates phenotype comparison, miRNA-mRNA modules, transcription-factor network remodelling, late anthocyanin-biosynthesis activation, flavonoid transport and pigment accumulation.

Together, the transcriptomic, small-RNA and metabolomic results support a working model for Pink leaf formation (Figure 7). Increased expression of late anthocyanin-biosynthesis and glycosylation genes coincided with anthocyanin-glycoside accumulation. Predicted miRNA associations may contribute to changes in DFR and TT12-like transcript abundance. Transcription-factor remodelling may provide an additional regulatory layer.

This model remains hypothetical because this study is based on correlative multi-omics data. Bulk leaf measurements may also average signals from green and Pink-purple cell populations. This study did not include target-cleavage assays, transient-expression tests or genetic perturbation. Reciprocal crosses and progeny-segregation analyses were also not performed.

Future work should validate the leading miRNA–mRNA candidates in independent biological samples. Spatial expression analysis could determine whether candidate transcripts are specifically enriched within coloured sectors. Target-cleavage, reporter and gene-perturbation experiments would be required to test causality. Segregating populations and progeny phenotyping are needed to determine the inheritance and stability of the variegated phenotype.

## 5. Conclusions

Integrated mRNA, miRNA and metabolomic analyses revealed that Pink-purple variegation in sweetpotato leaves was associated with coordinated changes in anthocyanin biosynthesis and flavonoid transport. The total relative flavonoid abundance in Pink leaves was 2.98 times that in CK leaves, with pronounced enrichment of petunidin, delphinidin and cyanidin glycosides. PAL, F3H, DFR, ANS and UFGT were also upregulated, consistent with enhanced anthocyanin biosynthesis during the middle and late stages of the pathway. The inverse expression patterns of iba_638_x1–G10133|TU16655 (DFR) and iba_730_x1–G3592|TU5891/G46236|TU74506 (TT12/TT12-like) highlighted these pairs as candidate regulatory modules. Together, these candidates may link anthocyanin production with pigment transport and accumulation. The identified genes and metabolites provide targets for functional validation and may inform the future development of sweetpotato germplasm with ornamental and potential functional-food value.

## Figures and Tables

**Table 1:** Differential expression of transcription factor families in Pink relative to CK.

Transcription Factor Family	Annotated Members	FDR < 0.05	FDR < 0.05 and |log_2_FC| > 1	Upregulated	Downregulated
MYB	79	23	20	8	12
bHLH	114	32	27	10	17
WD40/WD-repeat	104	28	22	11	11

## Data Availability

The original contributions presented in this study are included in the article/Supplementary Materials. Further inquiries can be directed to the corresponding author.

## Supplementary Materials

The following supporting information can be downloaded at: https://www.mdpi.com/article/10.3390/plants15152393/s1, Figure S1: Phenotypes and sampling groups used for RNA sequencing. Representative normal green-leaf sweetpotato plants and variegated sweetpotato plants were designated as CK and Pink, respectively. Three biological replicates were collected from each group for RNA sequencing; Figure S2: Sampling scheme and sample ID assignment for metabolomic analysis. Samples 1-1, 1-2, and 1-3 represent the purple leaf, pink leaf, and central variegated leaf, respectively; Table S1: Summary of quality-control results for the six small RNA sequencing libraries; Table S2: Comprehensive table of differentially expressed miRNAs between Pink and CK leaves.

## Abbreviations

The following abbreviations are used in this manuscript:

ANS	anthocyanidin synthase
bHLH	basic helix-loop-helix
CHI	chalcone isomerase
CHS	chalcone synthase
CK	normal green-leaf control
DBAT	10-deacetylbaccatin III-10-O-acetyltransferase
DFR	dihydroflavonol 4-reductase
F3H	flavanone 3-hydroxylase
FDR	false discovery rate
FC	fold change
FLS	flavonol synthase
LDOX	leucoanthocyanidin dioxygenase
MBW	MYB-bHLH-WD40 complex
miRNA	microRNA
mRNA	messenger RNA
PAL	phenylalanine ammonia-lyase
qPCR	quantitative polymerase chain reaction
RNA-seq	RNA sequencing
SPL	SQUAMOSA promoter-binding protein-like
TT12	TRANSPARENT TESTA 12
UFGT	UDP-glucose:flavonoid 3-O-glucosyltransferase
WD40	WD40-repeat protein
